# Supplementary material for: The Predictive Value of the Fibrinogen–Albumin-Ratio Index on Surgical Outcomes in Patients with Advanced High-Grade Serous Ovarian Cancer
Source: Cancers (Basel). 2024 Sep 27;16(19):3295. doi: 10.3390/cancers16193295 (PMC11476045; doi:10.3390/cancers16193295)
Supplement: Supplementary file 1 [file cancers-16-03295-s001.zip › cancers-3172840-supplementary.pdf]

Supplementary Materials

Table S1. Predictive value of fibrinogen and albumin alone in multivariable binary logistic regression model with the endpoint of complete tumor resection during primary cytoreductive surgery in advanced high-grade serous ovarian cancer patients.

	**Multivariable for Fibrinogen**		**Multivariable for Albumin**	
Parameter	p-value	OR (95% CI)	p-value	OR (95% CI)
FARI ≥ 11.06	-	-	-	-
CCI ≥ 3	0.797	0.89 (0.38 – 2.11)	0.614	0.80 (0.34 – 1.90)
CA125 ≥ 683.0kU/l	0.709	0.87 (0.43 – 1.79)	0.568	0.81 (0.39 – 1.67)
FIGO stage IIb- IIIc vs IV	0.321	1.75 (0.58 – 5.27)	0.419	1.56 (0.53 – 4.62)
ascites ≤ 50ml / 51 – 499ml / ≥ 500ml	0.068	1.43 (0.97 – 2.11)	0.134	1.35 (0.91 – 2.00)
fibrinogen ≥ 4.46g/L	0.005	2.71 (1.34 – 5.46)	-	-
albumin ≤ 39.9g/L	-	-	0.005	2.78 (1.36 – 5.84)

Preoperative fibrinogen and albumin are depicted for descriptive purposes only to contextualize the predictive value of the FARI. Considering the same significance threshold of $p<0.0042$ after applying a Bonferroni correction as compared to Table 2 (accounting for 7 univariate and 5 multivariable tests per model, respectively), observations are to be interpreted with caution. HGSOC, high-grade serous ovarian cancer; CCI, Charlson Comorbidity Index; FIGO, International Federation of Gynaecology and Obstetrics; OR, odds ratio; CI, confidence interval.

Table S2. a. Univariable and multivariable cox regression models with the endpoint of progression free survival in high-grade serous ovarian cancer patients.

	**Univariable**		**Multivariable**	
Parameter	p-value	HR (95% CI)	p-value	HR (95% CI)
FARI	< 0.001[a]	1.07 (1.04 – 1.10)	0.002[b]	1.06 (1.02 – 1.09)
CCI	0.079[a]	1.10 (0.99 – 1.22)	0.553[b]	1.05 (1.02 – 1.09)
CA125 levels kU/l	0.553[a]	1.00 (1.00 – 1.00)	-	-
FIGO tumor stage IIb - IIIc vs IV	0.033[a]	1.85 (1.05 – 3.25)	0.103[b]	2.03 (0.87 – 4.76)
complete tumor resection	< 0.001[a]	2.54 (1.78 – 3.61)	< 0.001[b]	2.25 (1.56 – 3.26)
time between surgery and first cycle of adjuvant chemotherapy (days)	0.243[a]	1.01 (0.99 – 1.03)	-	-

HGSOC, high-grade serous ovarian cancer; CCI, Charlson Comorbidity Index; FIGO, International Federation of Gynaecology and Obstetrics; HR, hazard ratio; CI, confidence interval. a univariable Cox-regression analysis. b multivariable Cox-regression analysis.

Table S2. b. Univariable and multivariable cox regression models with the endpoint of disease specific survival in high-grade serous ovarian cancer patients.

	**Univariable**		**Multivariable**	
Parameter	p-value	HR (95% CI)	p-value	HR (95% CI)
FARI	< 0.001[a]	1.07 (1.03 – 1.10)	0.001[b]	1.07 (1.03 – 1.11)
CCI	< 0.001[a]	1.22 (1.09 – 1.36)	0.006[b]	1.28 (1.07 – 1.52)
CA125 levels kU/l	0.987[a]	1.00 (1.00 – 1.00)	-	-
FIGO tumor stage IIb - IIIc vs IV	0.024[a]	2.02 (1.10 – 3.70)	0.897[b]	1.06 (0.42 – 2.71)
complete tumor resection	< 0.001[a]	2.16 (1.46 – 3.20)	0.003[b]	1.87 (1.24 – 2.81)
time to adjuvant chemotherapy (days)	0.097[a]	1.00 (1.00 – 1.00)	-	-
recurrence	< 0.001[a]	32.78 (4.61 – 233.22)	-	-

HGSOC, high-grade serous ovarian cancer; CCI, Charlson Comorbidity Index; FIGO, International Federation of Gynaecology and Obstetrics; HR, hazard ratio; CI, confidence interval. a univariable Cox-regression analysis. b multivariable Cox-regression analysis.
